# Phylogeography of the *Sinica* Group of Macaques in the Himalayas: Taxonomic and Evolutionary Implications

**DOI:** 10.3390/biology13100795

**Published:** 2024-10-04

**Authors:** Laxman Khanal, Xueyou Li, Asmit Subba, Sapana Ulak, Randall C. Kyes, Xue-Long Jiang

**Affiliations:** 1 Central Department of Zoology, Institute of Science and Technology, Tribhuvan University, Kathmandu 44618, Nepal; subbaasmit926@gmail.com (A.S.); sapanaulak999@gmail.com (S.U.); 2 Key Laboratory of Genetic Evolution and Animal Models, Kunming Institute of Zoology, Chinese Academy of Sciences, Kunming 650223, Yunnan, China; lixueyou@mail.kiz.ac.cn; 3 Departments of Psychology, Global Health, and Anthropology, Center for Global Field Study, and Washington National Primate Research Center, University of Washington, Seattle, WA 98195, USA; rkyes@uw.edu

**Keywords:** phylogeny, biogeography, species delimitation, *sinica* group, vicariance, dispersal hypothesis, Pleistocene climate

## Abstract

**Simple Summary:**

The taxonomy of the *sinica* group of macaques has been unresolved due to inconsistencies between their physical traits and genetic relationships. To clarify this taxonomic issue, we analyzed DNA from previously unsampled populations of the macaques in the Himalayas. Our results revealed that the *sinica* group consists of seven distinct species, rather than the previously considered six species as the two subspecies of Assamese macaques (*Macaca assamensis assamensis* and *M. a. pelops*) are separate candidate species with strong genetic variations. The study also identified Arunachal macaques (*M. munzala*) in the Shannan area of Xizang Zizhiqu in China. Multiple analyses revealed complex historical patterns of species dispersal and separation, mostly linked to climatic changes during the Quaternary period. We propose a new hypothesis about the *sinica* group’s evolutionary history.

**Abstract:**

Owing to the taxonomic incongruence between the morphological features and genetic relationships of the *sinica* group of macaques (genus *Macaca*), the taxonomy of this macaque group has remained inconclusive. We aimed to resolve the taxonomic quandary and improve our understanding of the historical biogeography of the group by including macaque DNA samples from previously unsampled areas in the Himalayas. We sequenced and analyzed three mitochondrial DNA loci [cytochrome b (CYTB), cytochrome oxidase subunit 1 (COI) and D-loop; 2898 bp] for sequence polymorphism, phylogenetics, species delimitation, and ancestral area reconstruction. We confirmed the occurrence of Arunachal macaque (*Macaca munzala*) on the southern slopes of the Eastern Himalayas in the Xizang Zizhiqu (Tibet Autonomous Region) of China. The results revealed that the *sinica* group of macaques is a parapatric species group composed of seven distinct species. Phylogenetic and species delimitation analyses revealed that the two previously considered subspecies of Assamese macaques (the eastern subspecies *M. assamensis assamensis* and the western subspecies *M. a. pelops*) are two distinct species. The eastern Assamese macaque is a sister species to the Tibetan macaque, whereas the western Assamese macaque and Arunachal macaque are the closest genetic sister species. The *sinica* group of macaques underwent five vicariance and seven dispersal radiations in the past, which mainly coincided with the Quaternary climatic oscillations between the late Pliocene and the late Pleistocene. By integrating our phylogenetic and ancestral area reconstruction results with findings from previous paleontological and molecular studies, we propose a robust hypothesis about the phylogeography of the *sinica* group of macaques.

## 1. Introduction

The extant macaques (genus *Macaca*, Mammalia: Primates: Cercopithecidae) have been grouped into seven species-groups based primarily on genital morphology, geographical distribution patterns, behavior, and genetics [1,2]. The *sinica* group of macaques was initially described based on male genitalia, the glans of which are subacute and sagittate-shaped on dorsal view [3]. This polytypic species group currently consists of six recognized species, namely, *Macaca assamensis*, *M. radiata*, *M. sinica*, and *M. thibetana*, and two recently described species, namely, *M. munzala* and *M. leucogenys* [4,5]. Another species in the group, *M. selai*, has been newly described as inhabiting the Sela mountain pass in the Eastern Himalayas [6]. However, its distinct species status has been questioned for multiple reasons, including morphological similarities and sympatry with *M. munzala* [7]. The *sinica* group is regarded as a monophyletic assemblage of parapatric species, possibly derived from *M. fascicularis-*like ancestors [8,9]. Macaques in this group have a moderately fragmented distribution, which might be associated with the two separate waves of radiation [10].

The taxonomy of the *sinica* group of macaques has remained inconclusive due to the considerable geographic and genetic variation among the species [7,11]. Furthermore, descriptions of new macaque species (primarily based on morphology) from the Eastern Himalayan region [4,5] have added to the confusion. Inconsistencies in the morphological taxonomy within the *sinica* group also exist for several reasons, including a lack of species-specific distinguishing morphological characteristics [7]. The major morphological characteristic used for distinguishing the *sinica* group of macaques is the sagittate glans penis. However, a circular glans penis has been observed in *M. leucogenys* [4]. Relative tail length is the next most widely used morphological index in macaque taxonomy. However, the mean values are much more similar, as is the overlap in their range [4,5,7,12]. Pelage color also varies widely among the species in the *sinica* group at different elevations and has even been observed to vary by season [13], as well as by age and sex of the individuals within a single group [7]. According to ecogeographical rules such as Bergmann’s and Allen’s rules, larger body sizes and shorter tails occur in macaque populations at higher elevations and latitudes as a part of adaptations to colder climates [12]. Morphological characteristics such as body weight, pelage color, and tail length could be attributed to the adaptational features of populations in different environments and thus may not be a suitable option for taxonomic delineation [7].

The *sinica* group of macaques has a disjunct distribution in South and Southeast Asia. They have a continental distribution except for *M. sinica*, which inhabits a fringing island, Sri Lanka [14]. *Macaca radiata* is distributed in southern India, with a northern distribution range believed to extend to the rivers of Godavari in the east and Tapti in the west [15]. The Assamese macaque is distributed in South and Southeast Asia, and its eastern subspecies (*M. a. assamensis*) is separated from its western subspecies (*M. a. pelops*) by the great bend of the Brahmaputra River [7]. The nominotypic/eastern subspecies of the Assamese macaque lacks clear information on the type locality and its holotype specimen is also missing [7]. The western subspecies, originally described as being from ‘eastern Nepal’, has a wide range of distribution along the Himalayas, with morphological variations among subpopulations [16]. The Himalayan region is populated by *M. munzala* [5,17,18,19], *M. leucogenys* [4,20,21], and *M. a. pelops*, including the Nepal population, which has been described to be morphologically and genetically unique [11,22,23,24,25]. Morphologically, *M. munzala* looks more similar to *M. a. assamensis*, but, based on genetics and geographical distribution, *M. munzala* is closer to *M. a. pelops* [7,11,26].

Unlike the ecogeographic segregation pattern of macaques proposed by Fooden [27], the zoogeography of macaques in the Himalayan region is more complicated [28]. The complex geography of the Himalayas creates diverse habitat types, and the macaque species found in this region are distributed from lowland tropical-type forests to montane forests with no distinct geographical boundaries, to our knowledge. Among the six currently recognized species in the *sinica* group, the IUCN Red List of Threatened Species has categorized three (*leucogenys*, *munzala*, *sinica*) as Endangered (EN) [29,30,31], one (*radiata*) as Vulnerable (VU) [32], and two (*assamensis* and *thibetana*) as Near Threatened (NT) [24,33]. All these species are facing rapid population decline, likely due to ongoing habitat loss and degradation. Despite being legally protected by national legislation in range countries, these species suffer from illegal hunting and retaliatory killings driven by crop raiding [17,24,34,35,36]. Such killings are detrimental to threatened macaques when local people are unable to distinguish them from the abundant nuisance species, the rhesus macaque [35]. Therefore, it is essential to explore the distribution of macaques from understudied areas and resolve their taxonomy so that we can better understand their evolutionary dynamics and formulate well-informed conservation plans.

Studies on the taxonomy of macaques are often limited by inadequate sampling, such as a lack of samples representative of the species’ entire distribution ranges, especially from the Himalayan region. As discussed above, morphology-based taxonomy (e.g., body size, coat color, tail length, facial structure, etc.) has limitations in species delineation. Nuclear DNA has been less informative in resolving taxonomic relationships among the species in the *sinica* group of macaques [10,11,26], which might be attributed to shorter lengths of the analyzed loci and a slow rate of mutations in nuclear DNA [37,38], and a lack of sufficient genetic differentiation in recently separated taxa. Additionally, hybridization and male-mediated genetic introgression also create obscured evolutionary relationships when the analysis is based on autosomal and Y chromosomal DNA [39]. Mitochondrial DNA (mtDNA) has higher rates of mutations than nuclear DNA [38] and could be more informative in high-resolution analyses of the evolutionary process of recently diverged taxa [40,41]. Because macaques exhibit female philopatry, the matrilineally inherited mtDNA could be more useful for testing their evolutionary relationship. However, the mtDNA marker lengths used in phylogenetic analysis and recent new species descriptions under the *sinica* group are often too short. For example, *M. munzala* was originally described based on morphological features and a phylogenetic analysis of fragments of mitochondrial cytochrome b (CYTB, 424/1140 bp) and D-loop (534/1090 bp) sequences [26]. More recently, *M. selai* was described with a phylogenetic analysis based on a segment of D-loop (460/1090 bp) sequences only [6]. Therefore, a phylogenetic analysis employing multiple loci from mitochondrial DNA and sampling from all known distribution ranges could be a reasonable way to determine the taxonomy of the *sinica* group of macaques. Therefore, this study aimed to determine the phylogenetic relationships, delineate the species, and better understand the historical biogeography of the *sinica* group of macaques in the Himalayas based on mtDNA sequences. We analyzed sequences from three mtDNA loci, a noncoding control region (D-loop, 1090 bp), and two protein-coding genes, CYTB (1140 bp) and cytochrome oxidase 1 (COI, 668 bp), from previously unsampled populations and corresponding sequences retrieved from GenBank. Comprehensive sampling, the inclusion of previously unsampled populations, and the use of multiple analytical approaches have provided better insight into the taxonomy and phylogeography of the *sinica* group of macaques.

## 2. Materials and Methods

### 2.1. Study Area and Sampling

**Figure 1:**
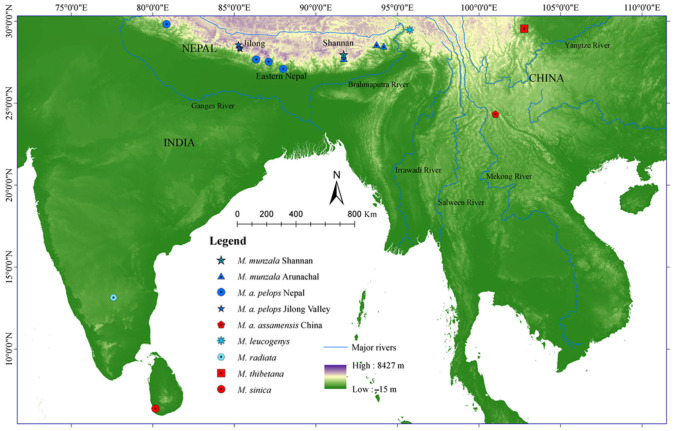
Study area with sampling locations of macaques in the Himalayas (Shannan, Jilong, and eastern Nepal) and locations of other macaque sequences retrieved from GenBank.

We conducted field surveys in the Eastern and central Himalayan regions and the southern areas of the Tibetan Plateau, covering previously unsampled populations. This included locations along the southern slopes of the Eastern Himalayas in the Shannan area of the Xizang Zizhiqu (Tibet Autonomous Region) of China, locations in the central Himalayas, including the Jilong Valley in Xizang, and parts of eastern Nepal (Figure 1).

#### 2.1.1. Shannan, Xizang, Eastern Himalayas

We conducted field surveys along the southern slopes of the Eastern Himalayas in Southwestern China’s Xizang Zizhiqu from 17 April to 10 May 2023. Population data were collected using camera traps and via line-transect sampling. A total of 57 camera traps were placed in the Shannan Forest area. Line-transect sampling involved the use of four transects, each 4–5 km in length, within elevational ranges of 2603–3783 m above sea level (asl). Our field survey and camera trap data identified three groups of macaques belonging to the *sinica* group from Shannan City in Southwestern China’s Xizang Zizhiqu. Morphological observations revealed that the three groups of macaques were similar to those of *M. munzala* (Figure S1). A total of nine fresh fecal samples were collected from the macaques in the three groups. Each sample was placed in a 20 mL sterilized tube containing 10 mL of RNAlater and stored at −20 °C for subsequent laboratory processing. Species identity was subsequently confirmed using mtDNA analysis.

#### 2.1.2. Jilong Valley, Xizang, Central Himalayas

We conducted similar field methods, employing camera traps and transect sampling, in the Jilong Valley of Southwestern China’s Xizang Zizhiqu in the central Himalayas from 22 May 2023 to 05 June 2023. The surveys were conducted within the elevational range of 1779–3750 m. Three groups of Assamese macaques with relatively long tails matching the morphology of *M. a. pelops* were observed. A total of 12 fresh fecal samples were collected from the macaques in these three groups, following the collection procedure described above.

#### 2.1.3. Eastern Nepal, Central Himalayas

Eastern Nepal is the typical locality for the western subspecies of Assamese macaques (*M. a. pelops*) [42]. We conducted field surveys in the Jhapa, Ilam, and Panchthar districts in the lower reaches of the Kanchenjunga Transboundary Landscape in eastern Nepal from 15 October to 12 November 2023. The sampling was conducted at elevations between 110 m asl and 2950 m asl. A total of 39 fresh fecal samples belonging to seven groups of Assamese macaques were collected. The samples were placed in sterilized plastic vials with 2 mL of lysis buffer [43]. Sterilized cotton swabs were used for the collection of fecal samples. A dry cotton swab was rolled along the surface of the feces and dipped in lysis buffer to recover epithelial cells from the feces. This process was repeated at least three times per fecal pellet. The fecal sample was turned over, and similar swabbing was performed using another cotton swab. The fecal samples were stored in lysis buffer at ambient temperature for further laboratory processing.

### 2.2. DNA Extraction, PCR Amplification, and Sequencing

We extracted genomic DNA from the fecal samples using the QIAamp DNA Stool Mini Kit (Qiagen, Germany), following the manufacturer’s protocols except for the following two modifications: 1) because the samples were collected in liquid media, 500 μL of each sample was used for DNA extraction, and 2) the final elution was performed in 50 μL of elution buffer. We amplified three mitochondrial loci, the control region/D-loop, cytochrome B (CYTB), and cytochrome oxidase subunit 1 (COI), using macaque-specific primer pairs (Table 1). The PCR volume of 25 μL contained 12.5 μL of 2 × PCR master mix (Taq PCR Master Mix, Qiagen, Germany), 0.5 μL of forward and reverse primers each, 11 μL of nuclease-free deionized water, and 0.5 μL of DNA template. The PCRs were carried out with initial denaturation at 94 °C for 5 min, 39 cycles of denaturation at 94 °C for 30 s, annealing at the respective temperatures (Table 1) for 30 s, and extension at 72 °C for 60 s, followed by a final extension at 72 °C for 10 min. The PCR products were tested in 1.5% agarose gels, and the successful amplicons were sequenced in both directions with the same pair of primers as for PCR amplification using a BigDye Terminator Cycle Kit v.3.1 (Invitrogen) on an ABI 3730XL sequencer (Applied Biosystems).

### 2.3. DNA Sequence Assembly and Analysis

#### 2.3.1. Sequence Assembly and Alignment

We assembled the DNA sequences using the SeqMan tool in DNASTAR Lasergene v7.1 [46]. Homologous sequences of the *sinica* group of macaques for the D-loop, CYTB, and COI loci were downloaded from GenBank (mitochondrial genomes were downloaded whenever available, and the three loci were retrieved) (Table 2). Because GenBank currently contains only the hypervariable region 1 (HVR1) segment sequence of *M. munzala* from the D-loop, the D-loop sequences for all other macaques were clipped to the corresponding segment for the genetic confirmation of *M. munzala* from Shannan, Xizang. For further phylogenetic analyses, the sequences for each locus were aligned using the ClustalW algorithm in MEGA 11 [47]. The final alignment contained 1090 bp for the D-loop, 1140 bp for the CYTB locus, and 668 bp for the COI locus.

#### 2.3.2. Phylogenetic Analysis and Divergence Time Estimation

We constructed the following maximum likelihood (ML) trees separately for the two alignments: (i) D-loop alignment to confirm the species for the macaques sampled from Shannan, Xizang; and (ii) CYTB + COI alignment (CYTB and COI, 1808 bp) for the detailed phylogeny among macaques. The ML analyses were carried out in the RAxML-HPC Blackbox 8.2.12 tool [48] with 1000 bootstrap replicates implemented in the CIPRES Science Gateway V 3.3 [49] online platform (https://www.phylo.org, accessed on 10 March 2024). To further validate the phylogenetic relationship among the *sinica*-group of macaques, a Bayesian inference (BI) tree was also constructed using concatenated protein-coding genes (CYTB and COI, 1808 bp) in BEAST v1.10.4 [50]. The best partitioning scheme and evolutionary models for the seven predefined partitions (Table S1) were determined using PartitionFinder v2.1.1 [51] and ModelFinder v2.2.0 [52], with the greedy algorithm and BIC criterion employed in Phylosuite v1.2.3 [53,54]. Two independent Markov chain Monte Carlo (MCMC) runs, each with 20 × 10^6^ generations, were run in BEAST v1.10.4 [50]. Samples were collected every 1000 generations, and the first four generations were discarded as burn-in. The convergence of the two MCMC chains between the two independent runs was ensured by <0.01 deviation of split frequencies. The MCMC convergence of each run was further assessed using TRACER v1.6 [55], with an effective sample size (ESS) >200.

Pairwise evolutionary distances among the *sinica* group of macaques in 1140 bp long CYTB gene sequences were calculated using MEGA 11 [47] using the Kimura-2 parameter (K2P) [56].

To estimate the divergence time among the macaques of *sinica* group, we constructed a time tree in BEAST v1.10.4 [50] using the concatenated CYTB, COI, and D-loop sequences (1140 bp + 668 bp + 1090 bp). The corresponding sequences of *Macaca sylvanus* and *M. mulatta* were used as outgroups. The estimated uncorrelated relaxed lognormal clock was selected as the clock model, and the Yule process and random starting tree were used as tree priors with 20 million MCMC generations and sampling every 5000 generations. Nodal calibrations were performed using the following two previously defined divergence dates [9,57]: (i) the most recent common ancestor of macaques at 5.5 million years ago (mya)—for this calibration, we established the prior using log-normal distribution with a mean in real space of 5.5 mya, an offset of 0, and a standard deviation of 0.05; (ii) the most recent proto-*sinica* fossil record at 3.2 mya, for which priors were established using log-normal distribution with a mean in real space of 3.2 mya, an offset of 0, and a standard deviation of 0.03.

#### 2.3.3. Molecular Species Delimitation

We performed four different molecular species delimitation analyses using the sequences of the CYTB, COI, and the D-loop. Nucleotide distance-based delimitation methods, namely ASAP (assemble species by automatic partitioning) [58] and ABGD (automatic barcode gap discovery) [59], were performed separately for the three markers. The ASAP analysis was performed using the K2P model in the program web interface (https://bioinfo.mnhn.fr/abi/public/asap, accessed on 10 March 2024), and ABGD analysis was performed using the Jukes–Cantor (JC69) model in the program web interface (https://bioinfo.mnhn.fr/abi/public/abgd/abgdweb.html, accessed on 10 March 2024). Two coalescent tree-based methods, namely, generalized mixed Yule coalescent (GMYC) [60] and multirate Poisson Tree Processes (mPTPs) [61], were used for species delimitation using the subset of the BI tree from concatenated sequences (2898 bp), using the *sinica* group of macaques as inputs. GMYC species delimitation analysis was performed using the default settings of the gmyc function of the R package splits 1.0.19 [62] in R studio [63]. The mPTP analysis was performed on the web interface (https://mptp.h-its.org/#/tree, accessed on 10 March 2024) with a single-rate Poisson tree processes method and a *p* value of < 0.001.

#### 2.3.4. Reconstruction of Ancestral Areas

We performed the ancestral area reconstruction for the *sinica* group of macaques using the program RASP (Reconstruct Ancestral State in Phylogenies) v.4.4 [64]. We tested the best model based on the lowest AIC and used statistical dispersal–vicariance analysis (S-DIVA) [65] for the reconstruction of ancestral areas. We used 5001 ultrametric trees and a BI consensus tree for the concatenated mtDNA dataset as inputs, and 100 random trees were used for each analysis. Based on the present distribution of the species, we used six areas as the distribution states for the *sinica* group of species: (A) South and Central China; (B) the central Himalayas; (C) southeastern Xizang; (D) the Eastern Himalayas; (E) Southern India; and (F) Sri Lanka. Integrating the S-DIVA analysis with previous paleontological [66] and molecular studies [10,26,67], we proposed a robust hypothesis about the phylogeography of the *sinica* group of macaques.

## 3. Results

### 3.1. Genetic Polymorphisms among the Sampled Populations

The D-loop sequences (n = 6) of *M. munzala* from Shannan, Xizang, identified three haplotypes (accession numbers PP874959–PP874961), whereas the CYTB sequences recognized two haplotypes (accession numbers PP884093–PP884094) (Table 3). *Macaca assamensis* sequences from the Jilong Valley (n = 12) yielded four haplotypes for both the D-loop and CYTB genes (accession numbers D-loop PP874955–PP874958, CYTB PP884089–PP884092). The D-loop sequences from *M. assamensis* of eastern Nepal (n = 36) identified nine unique haplotypes, and the CYTB sequences resulted in three haplotypes (accession numbers D-loop PP874946–PP874954, CYTB PP884086–PP884088). The COI sequences were less polymorphic, with two haplotypes in *M. munzala* from Shannan and three haplotypes each in *M. assamensis* from the Jilong Valley and eastern Nepal (accession numbers eastern Nepal PP868421–PP868422, Jilong PP868423–PP868425, Shannan PP868426–PP868427). The concatenated sequences (2898 bp) produced fifteen haplotypes in the Nepal population and eight haplotypes in the Jilong population of *M. a. pelops*, whereas four haplotypes were identified for the Shannan population of *M. munzala*. The highest haplotype and nucleotide diversities were observed in *M. assamensis* from eastern Nepal (Table 3).

### 3.2. Assignment of Study Populations to Known Species

**Figure 2:**
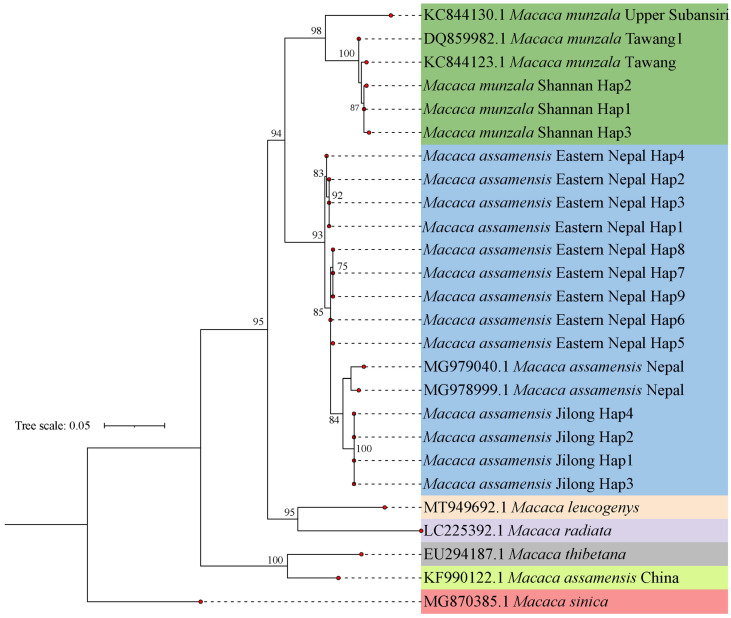
ML phylogenetic tree of the *sinica* group of macaques based on the HVR1 sequence (513 bp).

The maximum likelihood (ML) tree for the *sinica* group of macaques using HVR1 alignment depicted the closest affinity of the sequences from Shannan, Xizang, with the *M. munzala* sequences from Tawang and Upper Subansiri, which together formed a monophyletic clade with strong bootstrap support (Figure 2). Thus, the results of the HVR1 analyses support our morphology-based identification of macaques from Shannan as *M. munzala* and confirm the broad distribution of the species in the Eastern Himalayas. The next clade closest to the clade of *M. munzala* was the western subspecies of *M. assamensis* from Nepal and the Jilong Valley, Xizang. *Macaca leucogenys* and *M. radiata* formed another monophyletic clade, whereas *M. assamensis* from China and *M. thibetana* formed the fourth clade. *Macaca sinica* was the first to diverge from the ancestral stock of the group.

### 3.3. Phylogenetic Relationships and Evolutionary Distances among Macaques in sinica Group

**Figure 3:**
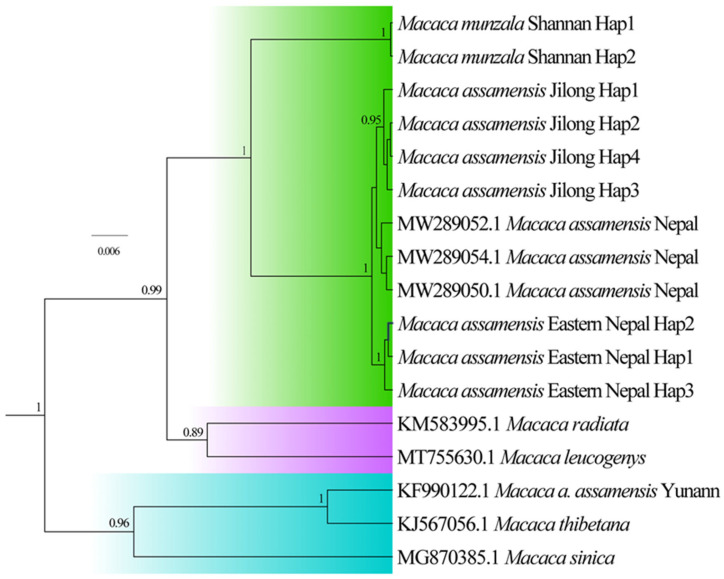
Bayesian inference (BI) phylogenetic tree of the *sinica* group of macaques based on concatenated protein-coding genes (CYTB and COI, 1808 bp).

To further verify the phylogenetic relationships among the *sinica* group of macaques in the Himalayas, we constructed ML and BI phylogenetic trees using the protein-coding mitochondrial CYTB (1040 bp) and COI (668 bp) genes. The BI (Figure 3) and ML (Figure S2) trees produced a similar tree topology, except for the sister relationship of *M. leucogenys* and *M. radiata* in the BI tree. This tree showed the closest evolutionary relationship of *M. munzala* from Shannan, Xizang, to the Nepal population of *M. assamensis*, but it was distant from *M. leucogenys* and *M. thibetana*, although the former (*M. munzala*) possessed a closer geographic distribution to the latter two congeners (*M. leucogenys* and *M. thibetana*) in the Eastern Himalayas and Xizang. The evolutionary distances estimated by the K2P model using CYTB sequences (Table 4) revealed the least genetic distance between *M. munzala* from Shannan, Xizang, and the population of *M. assamensis* from Nepal and the Jilong Valley (0.046).

### 3.4. Divergence Time Estimates and Species Delimitation

**Figure 4:**
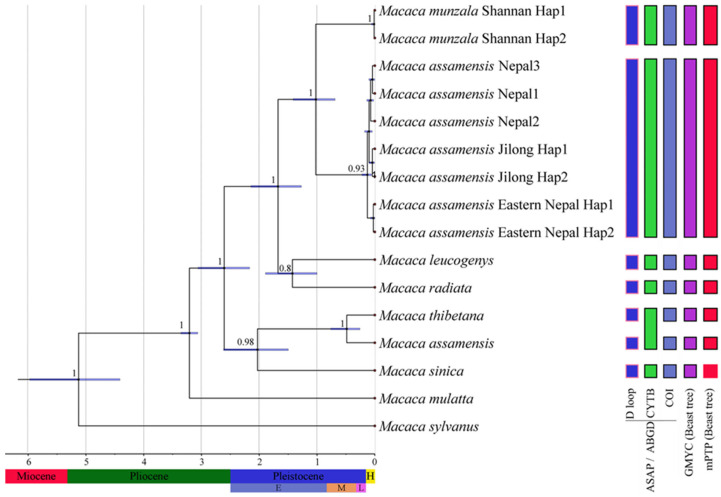
Ultrametric tree showing divergence time estimates for the *sinica* group macaques based on concatenated mtDNA sequences (2898 bp) using BEAST. The blue bars indicate the 95% highest posterior densities of divergence times; the nodal values represent Bayesian posterior probabilities. The results of species delimitation analyses (i.e., ASAP, ABGD, GMYC, and mPTP) are reported through vertical bars; the bar color indicates the different species delimitation analyses.

The ultrametric time tree based on concatenated mtDNA sequences (2898 bp) predicted that the most recent common ancestor of the *sinica* group of macaques occurred approximately 2.61 (95% HPD, 2.23–3.09) mya. At that time, the ancestral stock of *M. thibetana*, *M. sinica*, and *M. a. assamensis* (proto-*assamensis*–*sinica*–*thibetana* stock) diverged from the rest of the group. *M. sinica* diverged from the ancestral stock of the other two species approximately 2.03 mya. The ancestral stocks of *M. leucogenys* and *M. radiata* diverged from those of *M. munzala* and *M. assamensis* from Nepal approximately 1.68 mya. *Macaca munzala* and *M. a. pelops* split approximately 1.05 mya (Figure 4).

All four species delimitation analyses concurrently suggested that there are seven species in the *sinica* group of macaques except for the CYTB sequences of *M. assamensis* from China and *M. thibetana* in the ASAP and ABGD analyses (Figure 4). The *M. munzala* sequences from Xizang and *M. assamensis* sequences from Nepal and the Jilong Valley population also suggested these were distinct species.

### 3.5. Ancestral Area Inference for Sinica Group of Macaques

**Figure 5:**
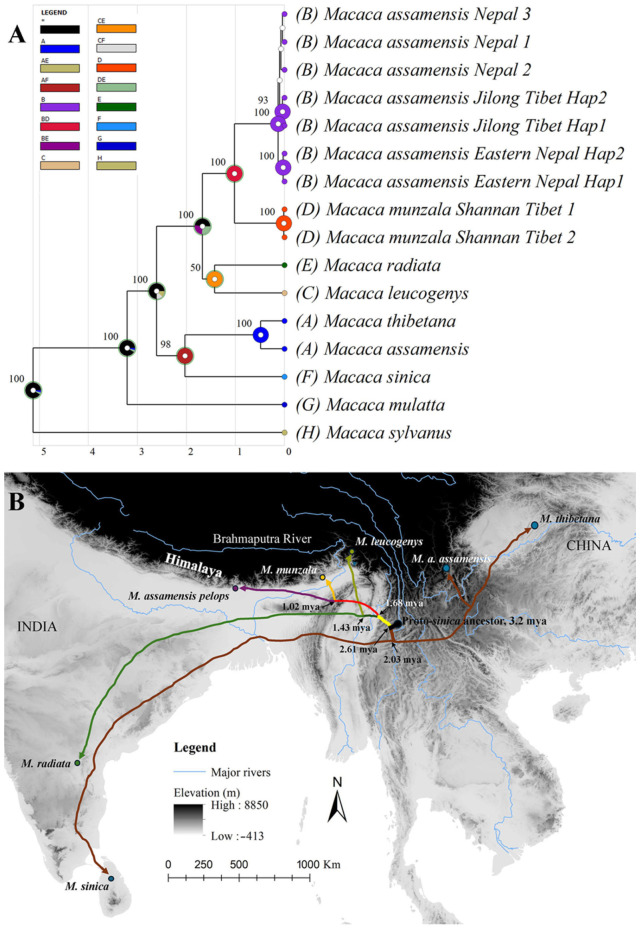
Ancestral area reconstruction and hypothetical dispersal of the *sinica* group of macaques. (**A**) S-DIVA output from RASP v.4.4 (the block letters in parenthesis represent distribution states for the *sinica* group of species as specified in the methods section); (**B**) a representation of the hypothetical divergence and dispersal of the *sinica* group of macaques based on phylogenetic, species delimitation, and ancestral area reconstruction analyses of mtDNA loci. The identification of the proto-*sinica* ancestor in Myanmar is based on paleontological evidence [64]. The geographical locations of the species represent the GPS points of the sequences used in this study. Dispersal routes are estimated and not precisely defined.

The S-DIVA in RASP v.4.4 revealed five global vicariance events and seven global dispersal events among the *sinica* groups of macaques used in the analysis (Figure 5A). The results suggested the speciation of all taxa by vicariance except for *M. assamensis* and *M. thibetana*, which underwent speciation by dispersal. Based on the phylogenetic analysis, divergence time estimation and S-DIVA, we proposed a hypothetical divergence and dispersal pattern of the *sinica* group of macaques, as shown in Figure 5B.

## 4. Discussion

The taxonomy of the *sinica* group of macaques has been a point of debate among researchers due to an incongruence in results when different morphological characteristics or genetic markers are used for analysis. This study assessed the evolutionary relationships among the *sinica* group of macaques by employing three mitochondrial DNA loci sequences and focusing on the species distributed in the Himalayas. We conducted comprehensive sampling from previously unsampled populations and integrated available data from other areas. Multiple approaches to phylogenetic and species delimitation analysis revealed that the *sinica* group of macaques is a polytypic group consisting of seven (as opposed to six) genetically distinct taxa/candidate species. The results indicated the distinct candidate species [68] status of the Assamese macaque from the Himalayas, which was previously considered the western subspecies (*M. assamensis pelops*). Furthermore, based on photographic evidence and phylogenetic analysis, we confirmed the distribution of *M. munzala* along the southern slopes of the Himalayas in Shannan City, Southwestern China’s Xizang Zizhiqu.

Three species of the *sinica* group, namely, *M. radiata*, *M. sinica* and *M. thibetana*, have distinct geographic ranges and have long been identified as distinct species; hence, there are no taxonomic disparities among primatologists studying these species. Based on the geographic range of distribution and relative tail lengths of *M. assamensis*, some researchers have concluded that *M. assamensis* can be divided into two subspecies: the eastern subspecies (*M. a. assamensis*) and the western subspecies (*M. a. pelops*) [69,70]. Others, however, have suggested that the western subspecies of the Assamese macaque represents a distinct species [11,23,25]. Additionally, the recently described *M. munzala* [5] and *M. leucogenys* [4], along with the previously considered western subspecies of Assamese macaque (*M. a. pelops*), are distributed in the Eastern and central Himalayan regions. Previous research focusing on the morphological characteristics used to describe these three taxa was not clear enough to distinguish them as separate species; hence, they have often been considered Assamese macaque complexes [7]. Phylogenetic analysis and multiple approaches for molecular species delimitation in the present study revealed that *M. munzala*, *M. leucogenys*, and *M. a. pelops* (from Nepal and Xizang) represent three distinct species. Hence, based on the mitochondrial DNA analysis in this study, the results suggest that the *sinica* group of macaques includes seven candidate species.

We believe that the population of Assamese macaques in the Himalayas, which was previously considered the western subspecies (*M. a. pelops*), is a distinct species and needs a comprehensive taxonomic description. The Assamese macaque haplotypes from the Jilong Valley in the Xizang Zizhiqu of China are genetically closer to the haplotypes from central Nepal. These phylogenetic relationships among the haplotypes of Assamese macaques in central Nepal and the Jilong Valley and estimates of divergence times support our earlier hypothesis [71] about the Pleistocene glacial refugia for the species in central Nepal during the Last Glacial Maximum (LGM) followed by their dispersal during the Holocene. The eastern subspecies of Assamese macaque (*M. a. assamensis*) forms a monophyletic clade with *M. thibetana*, whereas the western subspecies forms a separate clade with *M. munzala*, showing a polyphyletic origin of the two subspecies. A similar relationship indicating a polyphyletic origin of the species was described by Hoelzer, Hoelzer, and Melnick [8], based on mtDNA restriction site analysis, and by Khanal, Chalise, Fan, Kyes, and Jiang [11], based on multilocus phylogenetic analysis using both mtDNA and Y chromosomal gene sequences. The taxonomic descriptions of the two subspecies of Assamese macaques in the mid-19th century [42,72,73] were based on limited information, such as relying solely on morphological observations, localized geographical surveys, and a lack of reliable genetic data. Because these two subspecies have allopatric distributions [7,22,74], morphological dissimilarities [25], and genetic polyphyletic origins, they should be considered as two valid species in the *sinica* group.

The phylogeographic history of the *sinica* group of macaques involved multiple vicariance and dispersal events. Combining our results with the evidence from paleontological [66] and genetic studies [10,67], we propose a diversification and dispersal hypothesis for the *sinica* group of macaques. The first split from the ancestral stock of the *sinica* group occurred approximately 2.61 mya into the following two major groups: (i) the proto-*sinica*–*assamensis*–*thibetana* stock and (ii) the proto-*radiata*–*leucogenys*–*munzala-a. pelops* stock. This split might be attributed to the onset of major Northern Hemisphere glaciation during the late Pliocene and early Pleistocene. Our divergence time estimation aligns with other studies using mtDNA alone or with nuclear DNA [10,11]; however, this finding differs from the Y chromosomal inference of approximately 1.5–1.7 mya [75]. Contrary to the proposal of Chakraborty, Ramakrishnan, Panor, Mishra, and Sinha [26] about the late divergence of *M. sinica* from proto-*radiata*–*sinica* stocks, our results indicate that the *M. sinica* ancestors radiated during the first round of radiation as proto-*sinica*–*assamensis*–*thibetana* group. This genetic relationship among the three species (*assamensis*, *sinica*, and *thibetana*) is also supported by mitogenomic analysis [67]. The *M. sinica* ancestors split from the stock at approximately 2.03 mya and moved south–westward to their present-day distribution range in Sri Lanka, while another stock dispersed to Southeast Asia and China, later turning into two parapatric species, *M. assamensis* and *M. thibetana*. The second major ancestral stock moved westward, and the proto-*leucogenys*–*radiata* stock and proto-*munzala*–*a. pelops* stock diverged by approximately 1.75 mya. The proto-*leucogenys*–*radiata* stock split at approximately 1.43 mya, and the former may have been dispersed northward, while the latter dispersed southward to their present-day geographic range in Southern India. The proto-*munzala*–*a. pelops* stock might have diverged approximately 1.02 mya with the intensification of glaciation during the last glacial maximum (LGM) in the Himalayan region, which lasted approximately 20–18 thousand years ago [76,77]. The onset of glaciation might have created two allopatric populations; the river valleys in Northeast India provided refugia for the eastern population, whereas the Koshi and Gandaki River valleys in Nepal provided refugia for the western population [69]. These isolated populations might have evolved independently and acquired some level of genetic uniqueness in a short period, but still retained some morphological similarities. After the LGM, during the warmer Holocene, the founder populations of *M. munzala* and *M. a. pelops* might have experienced population expansion and geographic dispersal northward [71] in the Eastern and central Himalayas, respectively.

This study helps resolve the discrepancies in taxonomy and provides a better understanding of the evolutionary pattern of the *sinica* group of macaques in the Himalayas. It also could be helpful for our understanding of the evolutionary pattern of other mammals, including nonhuman primates such as Himalayan langurs (*Semnopithecus* spp.) and rhesus macaques (*M. mulatta*). Across the diverse habitat types of the Himalayas, macaques of *sinica* group show a diverse array of morphological characteristics, and, even within a group, there can be a high level of variation [7]. Therefore, detailed intraspecific genetic studies are essential for identifying evolutionarily significant units (ESUs) and management units (MUs). Despite our efforts to expand the sampling of macaques across the Himalayas, macaques from Northeast India remain underrepresented in genetic databases. The inclusion of macaque populations from Northeast India in the analysis further strengthened our understanding of their phylogeography. This study confirms the species status of the currently considered western subspecies of the Assamese macaques, along with other two recently described species (*M. munzala* and *M. leucogenys*) from the Himalayas. A comprehensive study employing ecological, behavioral, and genomic data is needed to further elucidate the macaques’ phylogeography.

## 5. Conclusions

Using three maternally inherited mtDNA loci for phylogenetic and species delimitation analyses, this study confirmed that the *sinica* group of macaques contains seven candidate species. The members of this group have a wide continental distribution in South and Southeast Asia and on the island of Sri Lanka. Like their complex distribution, the species in the *sinica* group had a composite phylogeographical pattern of radiation and dispersal coinciding with the Quaternary climatic oscillations. The Pliocene–Pleistocene climatic transition initiated the first round of radiation in the *sinica* group of macaques, leading to two major ancestral stocks. These stocks underwent a series of vicariance and dispersal cycles and Pleistocene glacial–interglacial cycles, especially the last glacial maximum (LGM), which caused the diversification of the Himalayan population of macaques into distinct species.

## Figures and Tables

**Table 1:** Primer pairs and PCR conditions for amplification of the studied loci.

Locus	Primer	Primer Sequence (5′–3′)	Annealing Temp.	Reference
D-loop	LqqF	TCCTAGGGCAATCAGAAAGAAAG	58 °C	[44]
	Saru5	GGCCAGGACCAAGCCTATTT		[45]
CYTB	CYTF	AACCATCGTTGTACTTCAAC	56 °C	[11]
	CYTR	TCTGGTTTACAAGGCCAGTG		[11]
COI	MCOIF	TCAACAAACCATAAAGACATTGG	55 °C	This study
	MCOIR	AGACTTCGGGGTGACCAAAGAATC		This study

**Table 2:** Homologous sequences of the *sinica* group of macaques retrieved from GenBank and used for phylogenetic analysis.

Species	GenBank Accession Numbers			
	D-Loop	CYTB	COI	
*Macaca assamensis* China *	KF990122.1	KF990122.1	KF990122.1	
*Macaca assamensis* Nepal **	MG978999.1, MG979040.1, PP874946–PP874958 ^#^	MW289050.1, MW289052.1, PP884086–PP884092 ^#^	PP868421.1–PP868425.1 ^#^	
*Macaca leucogenys*	MT755630.1	MT755630.1	MT755630.1	
*Macaca munzala*	DQ859981.1, DQ859982.1, KC844122.1, KC844123.1, PP874959–PP874961 ^#^	PP884093–PP884094 ^#^	PP868426.1–PP868427.1 ^#^	
*Macaca radiata*	KM583995.1	KM583995.1	KM583995.1	
*Macaca sinica*	MG870385.1	MG870385.1	MG870385.1	
*Macaca thibetana*	KJ567056.1	KJ567056.1	KJ567056.1	
*Macaca mulatta* ^#^	AY612638.1	AY612638.1	AY612638.1	
*Macaca sylvanus*	KJ567054.1	KJ567054.1	KJ567054.1	

* Eastern subspecies (*M. a. assamensis*), ** Western subspecies (*M. a. pelops*), ^#^ sequences generated during this study and submitted to GenBank.

**Table 3:** DNA polymorphisms and genetic diversity in the sampled populations of macaques from the Himalayas.

Species (Location)→ Locus↓		*M. munzala* (Shannan, Xizang)	*M. a. pelops (*Jilong, Xizang)	*M. a. pelops* (Eastern Nepal)
Samples (Sequences)	N	9 (6)	12 (12)	39 (36)
D-loop (1090 bp)	H	3	4	9
	Hd ± SD	0.600 ± 0.215	0.758 ± 0.081	0.903 ± 0.017
	π ± SD	0.0017 ± 0.0009	0.0014 ± 0.0002	0.0046 ± 0.0003
CYTB (1140 bp)	H	2	4	3
	Hd ± SD	0.533 ± 0.172	0.561 ± 0.154	0.686 ± 0.019
	π ± SD	0.001 ± 0.0003	0.0007 ± 0.0003	0.0008 ± 0.0001
COI (668 bp)	H	2	3	2
	Hd ± SD	0.333 ± 0.215	0.545 ± 0.144	0.413 ± 0.068
	π ± SD	0.0005 ± 0.0003	0.0012 ± 0.0003	0.0006 ± 0.0001
D-loop + CYTB + COI (2898 bp)	H	4	8	15
	Hd ± SD	0.867 ± 0.129	0.924 ± 0.057	0.937 ± 0.018
	π ± SD	0.0011 ± 0.0003	0.0011 ± 0.0002	0.0023 ± 0.0001

Note: N—number of samples (sequences), H—number of haplotypes, Hd—haplotype diversity, π—nucleotide diversity, SD—standard deviation, →—species name and locality, ↓—locus.

**Table 4:** Estimates of evolutionary distance (below diagonal) with standard error estimates (above diagonal) over CYTB sequence pairs among the *sinica* group of macaques using the K2P model.

	Species	1	2	3	4	5	6	7
1	*M. munzala* Xizang		0.0068	0.0087	0.0082	0.0103	0.0100	0.0116
2	*M. a. pelops* Nepal and Jilong	0.0457		0.0082	0.0079	0.0096	0.0094	0.0112
3	*M. radiata*	0.0734	0.0658		0.0075	0.0091	0.0088	0.0094
4	*M. leucogenys*	0.0740	0.0675	0.0649		0.0092	0.0091	0.0102
5	*M. a. assamensis* China	0.0983	0.0879	0.0855	0.0877		0.0041	0.0092
6	*M. thibetana*	0.0950	0.0847	0.0813	0.0837	0.0208		0.0086
7	*M. sinica*	0.1037	0.1003	0.0866	0.0989	0.0805	0.0722	

## Data Availability

The DNA sequences of the haplotypes generated during the study have been submitted to GenBank with the following accession numbers—Control Region (PP874946–PP874961); Cytochrome B (PP884086–PP884094); and COI (PP868421–PP868427).

## Supplementary Materials

The following supporting information can be downloaded at: https://www.mdpi.com/article/10.3390/biology13100795/s1, Table S1: Partitioning schemes and best-fit substitution models; Figure S1: Photographs of macaques observed and sampled during this study depicting morphological variation; Figure S2: Bayesian inference (BI) tree of concatenated CYTB and COI genes for the *sinica* group of macaques.
