# Supplementary material for: Phylogeography of the Sinica Group of Macaques in the Himalayas: Taxonomic and Evolutionary Implications
Source: Biology (Basel). 2024 Oct 4;13(10):795. doi: 10.3390/biology13100795 (PMC11504220; doi:10.3390/biology13100795)
Supplement: Supplementary file 1 [file biology-13-00795-s001.zip › biology-3222992-supplementary.pdf]

Phylogeography of the *sinica*-group of macaques in the Himalayas: Taxonomic and evolutionary implications

Laxman Khanal[1]*, Li Xueyou[2], Asmit Subba[1], Sapana Ulak[1], Randall C. Kyes[3], Xue-Long Jiang[2]*

[1]Central Department of Environmental Science, Institute of Science and Technology, Tribhuvan University, Kathmandu 44618, Nepal

[2]Key Laboratory of Genetic Evolution and Animal Models, Kunming Institute of Zoology, Chinese Academy of Sciences, Kunming, Yunnan 650223, China

[3]Departments of Psychology, Global Health, and Anthropology, Center for Global Field Study, and Washington National Primate Research Center, University of Washington, Seattle, WA 98195, USA

Correspondence: LK- laxman.khanal@cdz.tu.edu.np; XLJ- jiangxl@mail.kiz.ac.cn

Table S1. Partitioning schemes and best fit substitution models

The best partitioning scheme and evolutionary models for 7 pre-defined partitions were selected using PartitionFinder2 v2.1.1 (Lanfear et al., 2017), with greedy algorithm and BIC criterion.

Predefined partitions:

CYTB_pos1=1-1140\3;

CYTB_pos2=2-1140\3;

CYTB_pos3=3-1140\3;

COI_pos1=1141-1808\3;

COI_pos2=1142-1808\3;

COI_pos3=1143-1808\3;

D_loop=1809-2899;

Partitioning schemes and best-fit substitution models:

Subset partitions	Sites	Best model
P1: (CYTB_pos1, COI_pos1)	603	K80+I
P2: (COI_pos2, CYTB_pos2)	603	HKY+X
P3: (CYTB_pos3)	380	HKY+X
P4: (D_loop, COI_pos3)	1313	HKY+I+X

Lanfear, R., Frandsen, P. B., Wright, A. M., Senfeld, T., Calcott, B. (2016) PartitionFinder 2: new methods for selecting partitioned models of evolution for molecular and morphological phylogenetic analyses. Molecular Biology and Evolution. DOI: 10.1093/molbev/msw260

Figure S1. Photographs of macaques observed and sampled during this study

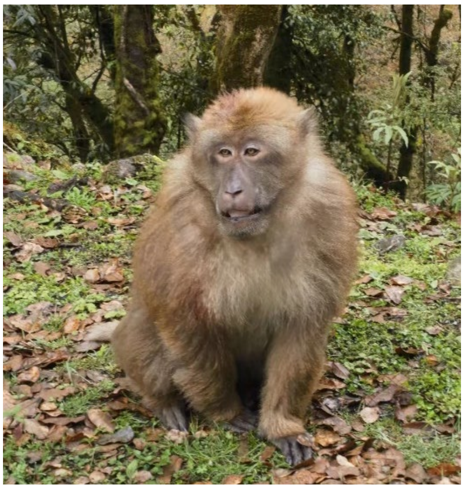

Photo 1. Adult male *M. munzala* from Shannan, Tibet

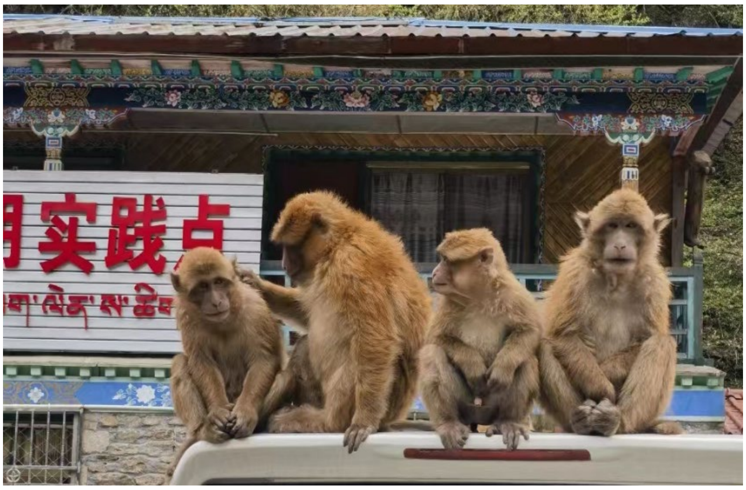

Photo 2. A group of *M. munzala* from Shannan Tibet

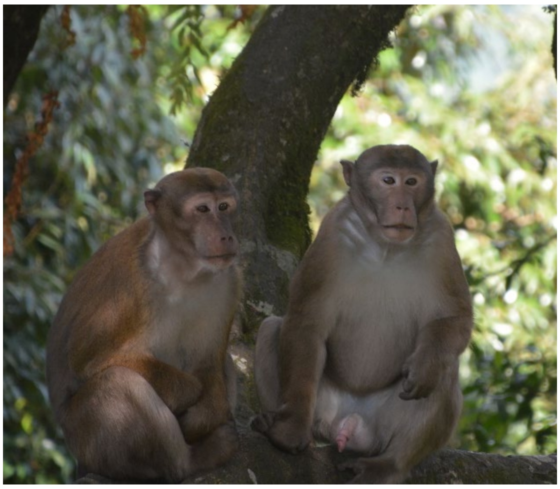

Photo 3. Two adult males of Nepal population of Assamese macaques from Ilam, eastern Nepal (sagittate glans penis is visible in one of them).

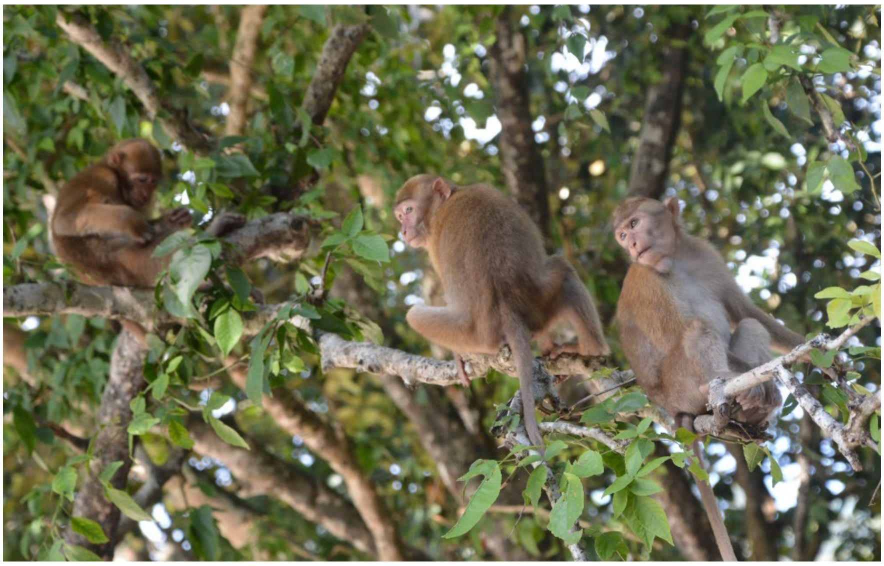

Photo 4. A group of Nepal population of Assamese macaques from Jhapa, eastern Nepal

Figure S2. Bayesian inference (BI) tree of concatenated CYTB and COI genes for the *sinica* group of macaques

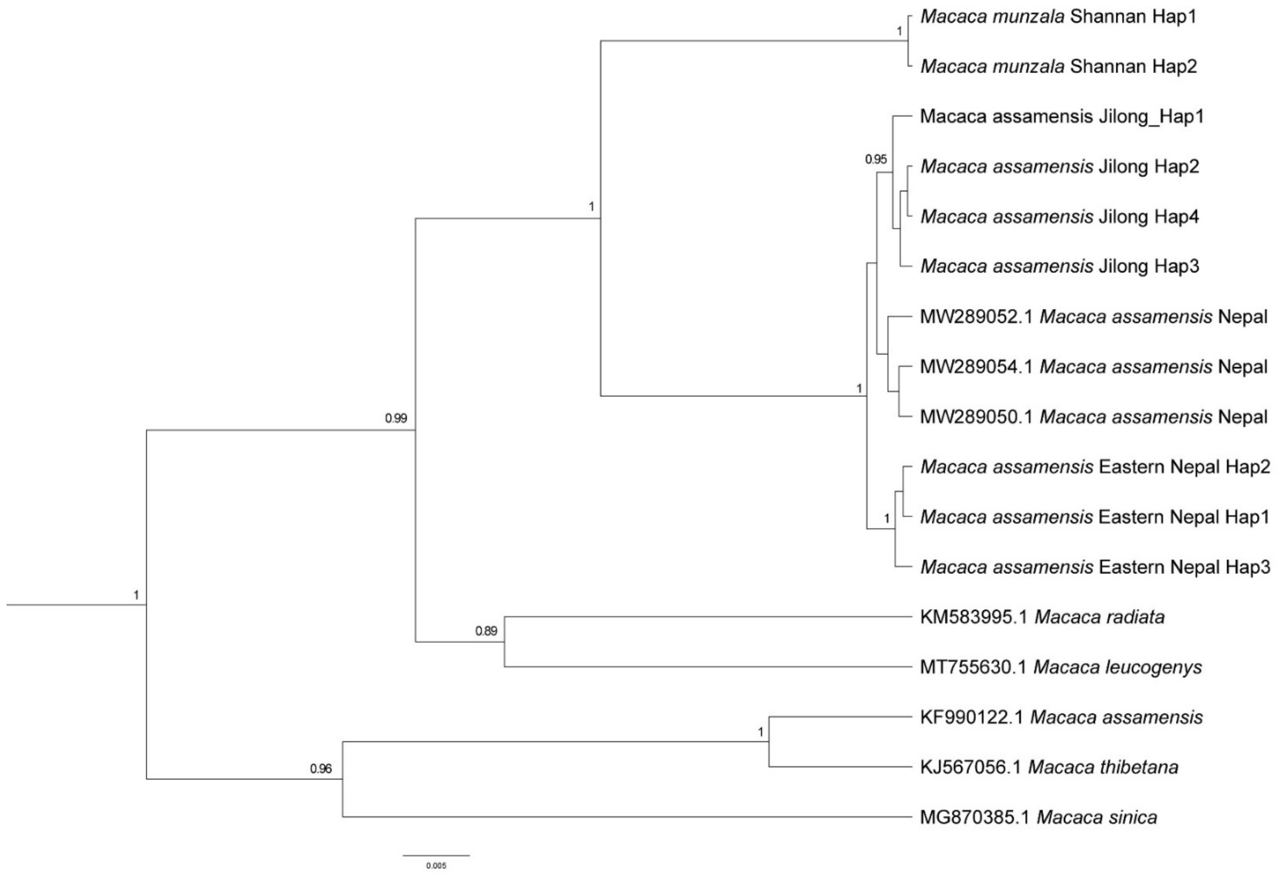

Figure S2. Bayesian inference (BI) phylogenetic tree among *sinica*-group of macaques based on concatenated protein-coding genes (CYTB and COI, 1808 bp).
